# Soluble pre-fibrillar tau and β-amyloid species emerge in early human Alzheimer’s disease and track disease progression and cognitive decline

**DOI:** 10.1007/s00401-016-1632-3

**Published:** 2016-10-21

**Authors:** David J. Koss, Glynn Jones, Anna Cranston, Heidi Gardner, Nicholas M. Kanaan, Bettina Platt

**Affiliations:** 1 School of Medical Sciences, University of Aberdeen, Foresterhill, Aberdeen, AB25 2ZD UK; 2 Department of Translational Sciences and Molecular Medicine, College of Human Medicine, Michigan State University, Grand Rapids, MI 49503 USA

**Keywords:** Alzheimer’s disease, Amyloid, Tau, Cognitive decline, Dementia, Pathology

## Abstract

Post-mortem investigations of human Alzheimer’s disease (AD) have largely failed to provide unequivocal evidence in support of the original amyloid cascade hypothesis, which postulated deposition of β-amyloid (Aβ) aggregates to be the cause of a demented state as well as inductive to tau neurofibrillary tangles (NFTs). Conflicting evidence suggests, however, that Aβ plaques and NFTs, albeit to a lesser extent, are present in a substantial subset of non-demented individuals. Hence, a range of soluble tau and Aβ species has more recently been implicated as the disease-relevant toxic entities. Despite the incorporation of soluble proteins into a revised amyloid cascade hypothesis, a detailed characterization of these species in the context of human AD onset, progression and cognitive decline has been lacking. Here, lateral temporal lobe samples (Brodmann area 21) of 46 human cases were profiled via tau and Aβ Western blot and native state dot blot protocols. Elevations in phospho-tau (antibodies: CP13, AT8 and PHF-1), pathological tau conformations (MC-1) and oligomeric tau (TOC1) agreed with medical diagnosis (non-AD cf. AD) and Braak stage classification (low, intermediate and high), alongside elevations in soluble Aβ species (MOAB-2 and pyro-glu Aβ) and a decline in levels of the amyloid precursor protein. Strong correlations were observed between individual Braak stages and multiple cognitive measures with all tau markers as well as total soluble Aβ. In contrast to previous reports, SDS-stable Aβ oligomers (*56) were not found to be reliable for all classifications and appeared likely to be a technical artefact. Critically, the robust predictive value of total soluble Aβ was dependent on native state quantification. Elevations in tau and Aβ within soluble fractions (Braak stage 2–3 cf. 0) were evident earlier than previously established in fibril-focused disease progression scales. Together, these data provide strong evidence that soluble forms of tau and Aβ co-localise early in AD and are closely linked to disease progression and cognitive decline.

**Electronic supplementary material:**

The online version of this article (doi:10.1007/s00401-016-1632-3) contains supplementary material, which is available to authorized users.

## Introduction

The original ‘amyloid hypothesis’ postulated that aggregated β-amyloid (Aβ) drives pathological mechanisms, results in the hyperphosphorylation of the microtubule-associated protein tau, and causes neurodegeneration and cognitive decline [29]. Accordingly, much of the early work focused on the deposition of Aβ as senile plaques (SPs) and their relationship to the emergence of tau pathology. This work was supported by genetic mutations identified in familial cases of AD (fAD), and has been greatly influential for both experimental research and therapeutic endeavours. Mutations in the amyloid precursor protein (APP) and the presenilin (PSEN) genes clearly demonstrated that dysfunctions in APP processing are causative of dementia in these cases. Yet, the contribution of specific cleavage products and protein aggregates to AD onset and progression as well as cognitive decline remains highly debated.

A prominent criticism of the amyloid hypothesis has been the lacking association of total plaque load with cognitive status, which is in contrast to the more robust and graded correlation of tau pathology to neuronal loss and symptomatic presentation. Indeed, the highly reproducible regional progression of tau containing neurofibrillary tangles (NFTs) and neuropil threads (NTs) provides the basis for neuropathological severity grading (Braak staging) [15]. Braak staging remains the most accepted post-mortem method of classifying AD progression, although amyloid pathology in the form of SPs and neuritic plaques must also be present to confer a true diagnosis of AD. Overall, NFTs are generally accepted as better indicators of mental impairment, clinical AD symptoms and neurodegeneration over plaque load [26, 53]. Both NFTs and NTs first emerge almost exclusively within the entorhinal cortex (EC) and hippocampal formation (transentorhinal stage, Braak stage 1–2), this pathology alone is associated with little or no cognitive impairment. Only when surrounding cortical and subcortical structures of the limbic system are affected (limbic stage, Braak stage 3–4) do individuals become symptomatic, and definitive symptoms develop fully only once this neuropathology engulfs the neocortex (neocortical stage, Braak stage 5–6).

For the conclusive neuropathological diagnosis of AD, an evaluation of Aβ depositions via complementary neurohistopathological schemes, such as the five SP-based Thal (Aβ) phases and CERAD (Consortium to Establish a Registry for Alzheimer’s Disease) staging must be made [12]. However, when considered independently, plaque deposition is less systematic cf. tau pathology and fails to correlate sufficiently with cognitive impairment [71]. Relative to NFTs, SP deposition follows a different spatial progression and is first observed within the neocortex [70]. Plaque numbers become more widespread reaching the EC and hippocampus long after the emergence of NFTs and NTs, with tau and amyloid pathologies only overlapping in advanced stages [73]. Such a spatial divide between the two pathological hallmarks makes it difficult to reconcile the proposed mechanistic and causative link between Aβ and tau, and cannot easily be incorporated into a common pathological scheme.

Despite this scenario, extensive research has focused on targeting amyloid-related pathologies for diagnostic purposes, e.g. based on PET amyloid imaging [78]. More recently, tau-based imaging markers have emerged with promising initial results [16]. Equally, biomarker research in cerebrospinal fluid (CSF) is on the hunt for multifactorial biomarkers, though assays with high specificity and sensitivity remain elusive. For example, a recent multi-centre study reported an age-independent occurrence rate of ~22 % of healthy controls (*n* = 1233) in which CSF Aβ levels were low enough to be classified as pathological, yet were negative for neurodegeneration and cognitively intact [76].

Beyond aspects related to disease diagnosis, neither Aβ plaques nor tau NFTs are likely the principle causative factor, as both of these forms of pathology are detected with considerable frequency in a subset of the non-demented population, referred to as non-demented high pathology controls (HPCs) [57]. Moreover, neurons can likely survive with NFT inclusions for several decades [56] and a separation of NFT formation and cognitive decline has been demonstrated in animal models [68]. Recent adaptations of the amyloid cascade hypothesis detail critical interactions with tau pathology, mechanisms of early synaptic loss and highlight central roles of soluble pre-fibril Aβ and tau species [55].

This shift away from insoluble pathology to potentially earlier emerging soluble Aβ and tau species required advanced biochemical techniques to establish an understanding for the progression of pre-fibrillar pathology. Significantly, a staging of Aβ pathology in line with disease progression has been proposed based on the abundance of low molecular weight Aβ species in cellular fractions, resolved by SDS-PAGE, and the degree to which these species demonstrate biochemical modifications [67]. The proposed stages correlate with established neuropathological grading scales, such as Braak and CERAD scores, to some degree, suggesting the potential for a stereotypical evolution of molecular pathology. However, associations with cognitive readouts and specific Braak stages were not included in this study. Therefore, a detailed evaluation of potentially pathology relevant soluble tau and Aβ species and their respective association with cognitive decline and disease progression within human cases identified as early and late-stage AD is still lacking.

Here, lateral temporal lobe tissue lysates (Brodmann area 21) from human AD cases and non-AD controls were quantified for phosphorylated, conformationally altered and oligomeric tau species alongside amyloidogenic changes in APP metabolism and levels of total, monomeric, oligomeric and pyro-glutamate (pyro-glu) modified Aβ species. Markers were assessed relative to several classifications, i.e. based on clinical diagnosis, grouped Braak stages (Br 0–2 cf. 3–4 cf. 5–6) as well as individual Braak stage, and correlated with each other as well as with cognitive decline. Our data demonstrate the earlier coincidence of soluble tau and Aβ and support the potential pathological role of both proteins early in the disease process of AD.

## Methods

### Tissue samples

Human temporal cortex samples (*n* = 46, middle temporal gyrus, Brodmann area 21) and corresponding metadata were supplied by MRC London Neurodegenerative Diseases Brain Bank, The Thomas Willis Oxford Brain Collection, The Manchester Brain Bank, The Newcastle Brain Tissue Resource, and The South West Dementia Brain Bank (see Table 1). All samples were received as 500 mg frozen blocks and were stored at −80 °C prior to use. Cases were de-identified but information was provided regarding age at death, post-mortem interval (PMI), cortical pH (where available), neuropathological assessment scores for Braak staging as well as CERAD neuritic plaque scores (where available) and cognitive scores for Mini Mental State Exam (MMSE) as well as global, memory and sum of box (SOB) scores established by the clinical dementia rating system (CDR). For full information regarding individual cases and neuropathological observations see Supplementary Table S1.

**Table 1:** Demographic data of human cohort and categories for data pooling

Categories	Braak stage	CERAD range	*N*	Male (%)	Age range (years)	Mean age (years)	PMI range (h)	Mean PMI (h)	pH range	Mean pH
**Diagnosis**										
Non-AD	0–3	C0–C2	27	44.4	74–103	86.04 ± 1.4	11–101	44.91 ± 5.2	5.4–6.9	6.25 ± 0.09
AD	4–6	C1–C3	19	60	71–90	83.58 ± 1.15	20–87	46.58 ± 4.9	6.01–6.9	6.34 ± 0.08
**Grouped Braak stages**										
Low	0–2	C0–C1	18	50	74–103	85.28 ± 1.88	11–92	40.67 ± 6.2	5.96–6.7	6.23 ± 0.08
Intermediate	3–4	C0–C3	14	42.9	77–95	85.86 ± 1.45	13.5–101	51.54 ± 6.9	5.4–6.9	6.22 ± 0.16
High	5-6	C1–C3	14	66.7	71–90	83.86 ± 1.39	20–78	46.00 ± 5.7	6.09–6.9	6.38 ± 0.08
**Braak stage**										
	0	C0	3	100	74–78	76.67 ± 1.33	11–56	30 ± 13.45	6.0–6.1	6.05 ± 0.05
	1	–	0	–	–	–	–	–	–	–
	2	C0–C1	15	40	74–103	87 ± 1.96	12–92	42.8 ± 7	5.96–6.7	6.32 ± 0.09
	3	C0–C2	9	33.3	78–95	87.56 ± 1.71	13.5–101	53.4 ± 9.5	5.4–6.9	6.2 ± 0.26
	4	C2–C3	5	40	77–88	82.8 ± 2.22	26–78	48.2 ± 10.6	6.01–6.8	6.24 ± 0.19
	5	C1–C3	6	83.3	82–88	83.8 ± 1.2	22–78	50.5 ± 10.1	6.09–6.6	6.28 ± 0.09
	6	C3	8	55.6	71–90	83.89 ± 2.12	20–69	42.6 ± 6.9	6.3–6.9	6.5 ± 0.14

CERAD neuritic plaque score, number of cases, gender composition (in  %), age, post-mortem interval (PMI) in hours (h) and frontal cortical pH of human cohort, classified according to medical diagnosis, grouped Braak stages and individual Braak stage. Mean values are indicated ± SEM

To serve as positive and negative immunoblot controls, murine forebrain samples from several transgenic strains were used (12-month old wild-type mice (C57/BL6) and 12-month-old bigenic fAD mice, as previously described [39]). Brain lysates from 12-month-old BACE1^−/−^ mice were a generous gift from Prof Michael Ashford (see [54]). Mice were housed and sacrificed in accordance with UK Home Office regulations, University European Directive on the Protection of Animals used for Scientific Purposes (2010/63/EU) and the Animal (Scientific Procedures) Act 1986.

### Immunoblot quantification of AD markers

Quantification of Aβ and tau markers was based as far as possible on near-native state preparations to limit the degree of sample manipulation required. Therefore, most markers were assessed by dot blots, with the exception of Western blots used for 6E10 and BACE1 antibodies to ascertain detection of single and relevant protein species. The oligomeric tau antibody TOC1 was also validated in Western blots to confirm detection of the previously reported oligomeric species, before samples were fully characterised via dot blots.

### Brain lysate preparation

100 mg of frozen cortical tissue was homogenised in ~1:10 (w/v) Igepal (Sigma, Dorset, UK) based lysis buffer (in mM: 20 HEPES, 150 NaCl, 0.1 EDTA, 1 % Igepal: pH = 7.6). All buffers were supplemented with complete protease inhibitors (Roche) and PhosStop tablets (Roche). The use of the non-ionic, non-denaturing Igepal, which is chemically indistinguishable from the widely used ‘Nonidet P-40’, ensured adequate lysis of plasma, endoplasmic and Golgi but not nuclear membranes, and prevented aggressive solubilisation of large aggregates [33]. Following manual homogenisation, samples were spun (13,000*g*, 4 °C, 20 min), supernatants separated from pellets, aliquoted and stored at −80 °C. For the determination of aggregated non-soluble protein pathology, Igepal derived pellets were re-suspended in excess Igepal buffer (1 ml) homogenised via repeated aspiration with a 1 ml pipette tip, briefly vortexed and spun (13,000*g*, 4 °C, 20 min). Following removal of the supernatants, the process was repeated. Resulting pellets were subsequently re-suspended in 1:1 (w/v) 70 % formic acid, incubated overnight at 4 °C with continuous agitation before a final spin (18,000*g*, 4 °C, 20 min) to yield the collected supernatant, which was stored at −80 °C until use.

### SDS-PAGE electrophoresis and protein transfer

Soluble lysates were adjusted for protein concentration (3 µg/µl) as per bicinchoninic acid colorimetric protein assay (BCA, Sigma). For standard denaturing SDS-PAGE electrophoresis, samples were mixed with lithium dodecyl sulphate (LDS, Nupage, Thermo Fisher Scientific, Paisley, UK) and dithiothreitol (15 mM DTT, Sigma) before being boiled for 10 min at 70 °C. For the identification of oligomeric tau species, samples were treated as above, except without DTT or boiling prior to gel loading. Insoluble samples were first mixed with 4 volumes of neutralising buffer (2 M Tris + 2 M NaH_2_PO_4_) before the addition of LDS and DTT.

Samples (30 µg/lane for soluble and 10 µl/lane for neutralised insoluble samples) were separated on 4–12 % Bis–Tris gels (Nupage, Thermo Fisher) under variable conditions dependent on protein target. For Aβ detection, proteins were separated for 35 min at 200 V constant voltage in MES buffer, transferred to 0.2 µm nitrocellulose membranes via IBlot (Nupage, Thermo-Fisher), and boiled for 3 min in 0.01 M Dulbecco’s Ca^2+^ and Mg^2+^ free phosphatase buffered saline (VWR International, Leicestershire, UK). For tau blots, electrophoresis was conducted for 45 min at 200 V in MOPS buffer and transferred to 0.45 µm nitrocellulose membranes via wet transfer conditions (1 h, 25 V constant).

### Solubility and protein extractions

The specific extraction of non-fibrillar proteins from human tissue post-mortem was confirmed in a series of comparative Western blots (Supplementary Figure 1). Total tau, visualised by the HT-7 antibody, demonstrated the selective isolation of tau within well-defined protein bands following Igepal lysis. In contrast, subsequent treatments of the remaining insoluble pellet with 70 % formic acid presented a smear of HT-7 immunoreactivity. This was only evident in AD cases and is indicative of highly post-translationally modified and aggregated tau species as found in paired helical filaments and NFTs. Similarly, limited solubilisation of amyloid species was observed via Igepal lysis, when compared to that isolated via formic acid from the pellet (see Supplementary Figure 1).

### Native state immunoblots

To ensure the preservation of potentially heat- and conformation-dependent epitopes, dot blots were conducted for a number of Aβ and tau pathological markers. Igepal soluble lysates were adjusted to 2 µg/µl with distilled H_2_O (concentration determined with BCA assay, as above) and directly dotted onto 0.2 µM nitrocellulose membranes (5 µl/samples, 10 µg/dot), which were allowed to dry prior to further processing. In a subset of experiments, designed to determine the heat-sensitivity of specific epitopes, lysates were heated (70 °C for 10 min) prior to being dotted.

### Blocking and antibody detection

All immunoblots were washed in 0.05 % Tween 20 (Sigma) containing Tris-buffered saline (TBST, in mM; 50 Trizma base, 150 NaCl, pH = 7.6) and blocked for 1 h at RT in 5 % milk powder containing TBST prior to overnight incubations at 4 °C with primary antibodies (see Supplementary Table 2) under continuous agitation. Antigen–antibody coupling was visualised using appropriate secondary antibodies conjugated to horseradish peroxidase (goat anti-mouse IgG or IgM, goat anti-rabbit IgG; 1:5000, Merk Millipore, Watford, UK) and enhanced chemiluminescence (1.25 mM Luminol, 30 µM coumaric acid, 0.015 % H_2_O_2_). All steps were followed by multiple washes in TBST. Processed blots were subsequently stained for measurements of total protein using Coomassie total protein stain (as described previously [65]). Images were captured via Vilber-Fusion-SL camera (Vilber, Eberhardzell, Germany) at 8-bit for illustration and 16-bit for analysis.

To ensure specificity of the detected signal, appropriate secondary antibody control dot blots were conducted, in which the primary antibody was excluded. For all cases, no detectable immunoreactive signal was observed for either IgG or IgM secondary antibody isotype in images captured following exposure times of up to 3 min, which was the maximal exposure time required for signal detection in this study (data not shown).

### Immuno-quantification

Immunoreactivity was quantified from 16-bit digitised images using ImageJ (Ver 1.47, NIH, USA) based on area under curve (AUC) measures normalised to total protein load established via AUCs for Coomassie processed blots. Immunoblot intensity data were normalised within blots using total protein adjusted values and expressed relative to appropriate control groups (outlined below), before being pooled across blots.

### Cohort stratification

To determine the relationship of investigated markers to the neuropsychological and pathological processes of AD, cases were grouped into several classifications as follows (see also Table 1):

1. Post-mortem confirmed clinical diagnosis, essentially equating to non-AD cases (=Braak (Br) 0–3) and AD (=Br 4–6).
2. Grouped Braak stages: low, intermediate and high severity of Braak pathology (Br 0–2, Br 3–4 and Br 5–6, respectively).
3. Individual Braak score: cases listed per individual stages and normalised to a single Braak score (Br 2).

### Statistical analysis

Statistical analysis was performed using Prism (V.6, GraphPad). Data were subject to Shapiro–Wilk test for normal distribution prior to statistical analysis of significance between groups. Individual comparisons were analysed using either a Student’s two tailed *t* test or Mann–Whitney if determined non-parametric. Multiple group comparisons were established via 1-way analysis of variance (ANOVA) or the non-parametric Kruskal–Wallis ANOVA, if reported as significant, selected pair comparisons were conducted via post hoc Bonferroni or Dunn’s tests, respectively. Analysis of covariance (ANCOVA) was employed to control for PMI and tissue pH (Minitab 17). Correlation analysis was conducted using Spearman’s ranks correlation, which does not assume normality of data sets. Having established either positive or inverse correlation of markers in relation to Braak stages, individual Braak stages were probed for significant deviation from Braak 0 via a one-tailed* t* test. For all tests, *p* < 0.05 was taken as significant with subsequent levels of statistical reliability reported for *p* < 0.01, *p* < 0.001 and *p* < 0.0001.

## Results

### Demographic data and cognitive scores

**Fig. 1:**
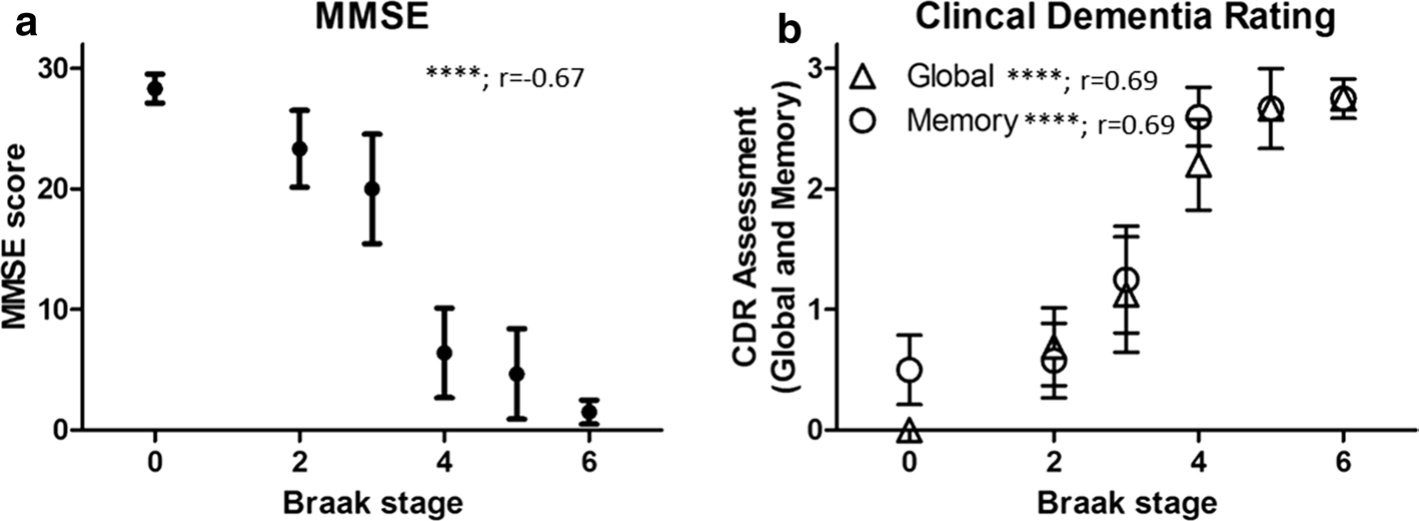
Cognitive performance during disease progression. Assessment scores from ‘Mini Mental State Exam’ (MMSE, **a**) as well as global and memory scores from the ‘Clinical Dementia Rating’ (CDR, **b**) are correlated with disease progression based on Braak stage. Significances (*p*) and Spearman’s correlation index (*r*) are provided within each graph. Data are expressed as mean ± SEM, ****p < 0.0001

Prior to the investigation of pathological markers, biographical data and post-mortem interval were investigated for potentially confounding factors with regards to pathological measures. Independent of analytical classification no significant difference in age, PMI or frontal cortical pH was determined between groups (Table 1). With the exception of Braak stage 0 (100 % male) and Braak stage 5 (83.3 % male), the cohort was reasonably balanced for gender. Scores of cognition as established by the MMSE (Fig. 1a, *r* = −0.67, *p* < 0.0001, *n* = 42), CDR global (Fig. 1b, *r* = 0.69, *p* < 0.0001, *n* = 43), CDR memory (Fig. 1b, *r* = 0.69, *p* < 0.01, *n* = 43) and CDR sum of box scores (data not shown, *r* = 0.55, *p* < 0.001, *n* = 33) closely followed Braak stage progression, but not age (data not shown).

### Quantification of tau pathology

**Fig. 2:**
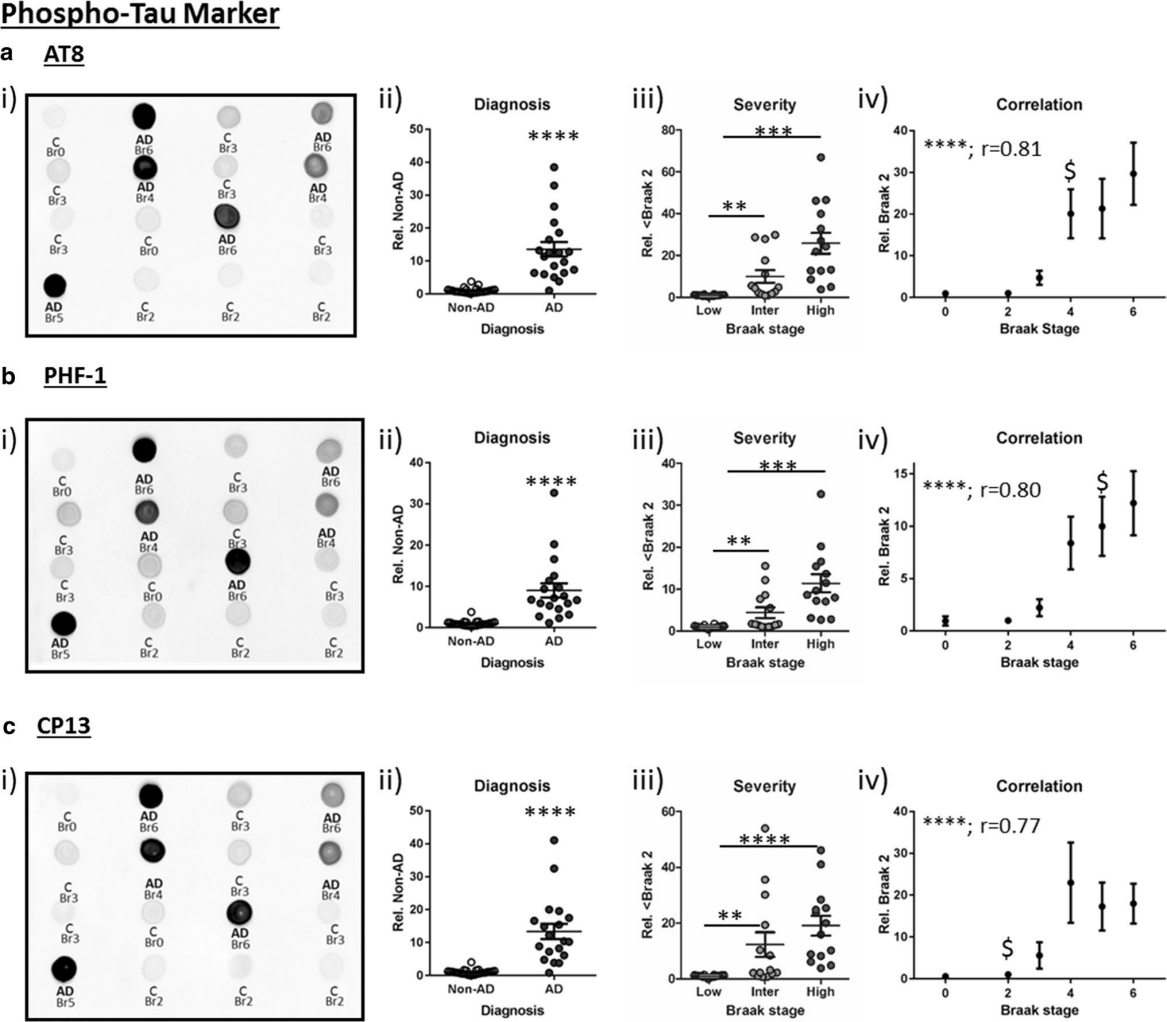
Phospho-tau pathology. Dot blots for **a** AT8, **b** PHF-1, **c** CP13 phospho-tau immunoreactivity. Cases within example dot blots are labelled for diagnosis [non-AD control cases (‘*C*’) and *AD*] and Braak stage (Br). Analyses stratified for ii) diagnosis, iii) severity (Low: Br 0–2, Intermediate (Inter): Br 3–4 and High: Br 5–6), and (iv) correlation with individual Braak stages are shown. Statistical outcomes are depicted as **p < 0.01, ***p < 0.001, ****p < 0.0001 and Spearman’s correlation (*r*). The earliest Braak stage at which immunoreactivity was significantly elevated from Braak 0 is also indicated ($)

Initially, phospho-tau pathology was investigated via immunoblots of soluble lysate, established to selectively extract non-fibrillar protein species (see Supplementary Figure 1). The phospho-specific antibody AT8 recognises tau phosphorylated at serine 199, serine 202 and threonine205 residues (p-ser199/ser202/thr205) [9, 27] and is commonly employed by pathologist for post-mortem confirmation of AD diagnosis and for the staging of tau pathology according to Braak stage. Under dot blot conditions, which negate alterations in the electrophoretic mobility of tau between samples, due to different degrees of protein phosphorylation [17] and allows for quantification of total tau species phosphorylated at each investigated epitope, AT8 immunoreactivity was strongly enhanced in AD diagnosed cases, reporting a ~14-fold increase compared to non-AD samples (Fig. 2a i + ii, *p* < 0.001). A graded increase in AT8 phospho-tau was also observed across grouped Braak stages (Fig. 2aii, low: Br 0–2, intermediate: Br 3–4, high: Br 5–6, *p* < 0.0001). Post hoc analysis demonstrated significant elevations between low and intermediate (*p* < 0.01) and low and high classifications (*p* < 0.0001), yet not between intermediate and high (*p* > 0.05). In accordance with the use of AT8 for the histopathological classification of tau pathology, dot blot quantification of soluble AT8 phospho-tau demonstrated a strong correlation with Braak staging (Fig. 2aiii, *r* = 0.81, *p* < 0.0001), initial elevations of AT8 phosphorylation being reported as significant from Braak 0 at Braak 4 (*p* < 0.05), which is in line with Braak stage progression for Brodmann area 21, reportedly affected at Braak stage 4 [2].

Tau phosphorylation was further investigated using two other commonly employed phospho-tau antibodies, PHF-1 (Fig. 2b, p-ser396/ser404) and CP13 (Fig. 2c, p-ser202). As for AT8, PHF-1 and CP13 reactivity was increased in AD compared to non-AD cases (Fig. 2bii + cii, 9- and 13-fold increase for PHF-1 and CP13, respectively, *p* < 0.0001 for both). Both PHF-1 and CP13 levels also increased with pathology severity (Fig. 2biii + ciii, *p* < 0.0001), reporting elevations within intermediate and high Braak stages compared to low pathology cases. Also, strong correlations with individual Braak stages were observed with PHF-1 and CP13 (Fig. 2biv + civ, *r* = 0.80, *p* < 0.0001 for PHF-1 and *r* = 0.77, *p* < 0.0001 for CP13). For the three phospho-epitopes, CP13 changes were detected earliest (Br 0 cf. Br 2: *p* < 0.05) and PHF-1 latest (Br 0 cf. Br 5, *p* < 0.05). Phospho-tau dot blots were validated for specificity by means of correlation of immunoreactivity with traditional Western blots in a subset of cases for PHF-1 (*r* = 0.79, *p* < 0.0001, *n* = 18, data not shown). A significant effect of Braak stage (*F*_(5,80)_ = 10.47, *p* < 0.0001) and epitope (*F*_(2,80)_ = 8.61, *p* < 0.001) and an interaction (*F*_(10,40)_ = 2.43, *p* < 0.05) was reported when comparing the phospho-epitope specific antibodies, likely due to the variable magnitude of hyperphosphorylation and differential antibody affinities apparent between Braak stages 2–4. Nevertheless, further analysis demonstrated a robust effect of subject matching, indicating those cases with high AT8 staining also demonstrated high levels of CP13 and PHF-1 staining. This is perhaps unsurprising given the similarity of dot blot immunostaining between epitopes (see example blots in Fig. 2).

### Conformational and oligomeric tau

Although hyperphosphorylation is the most extensively studied aspect of tau pathology, several additional protein modifications offer further key parameters in disease pathology. Conformational changes in the natively unfolded structure of the tau protein, leading to an interaction of the N-terminal domain with the microtubule binding domain (MTBD), may be closely modulated by phosphorylation status [81].

**Fig. 3:**
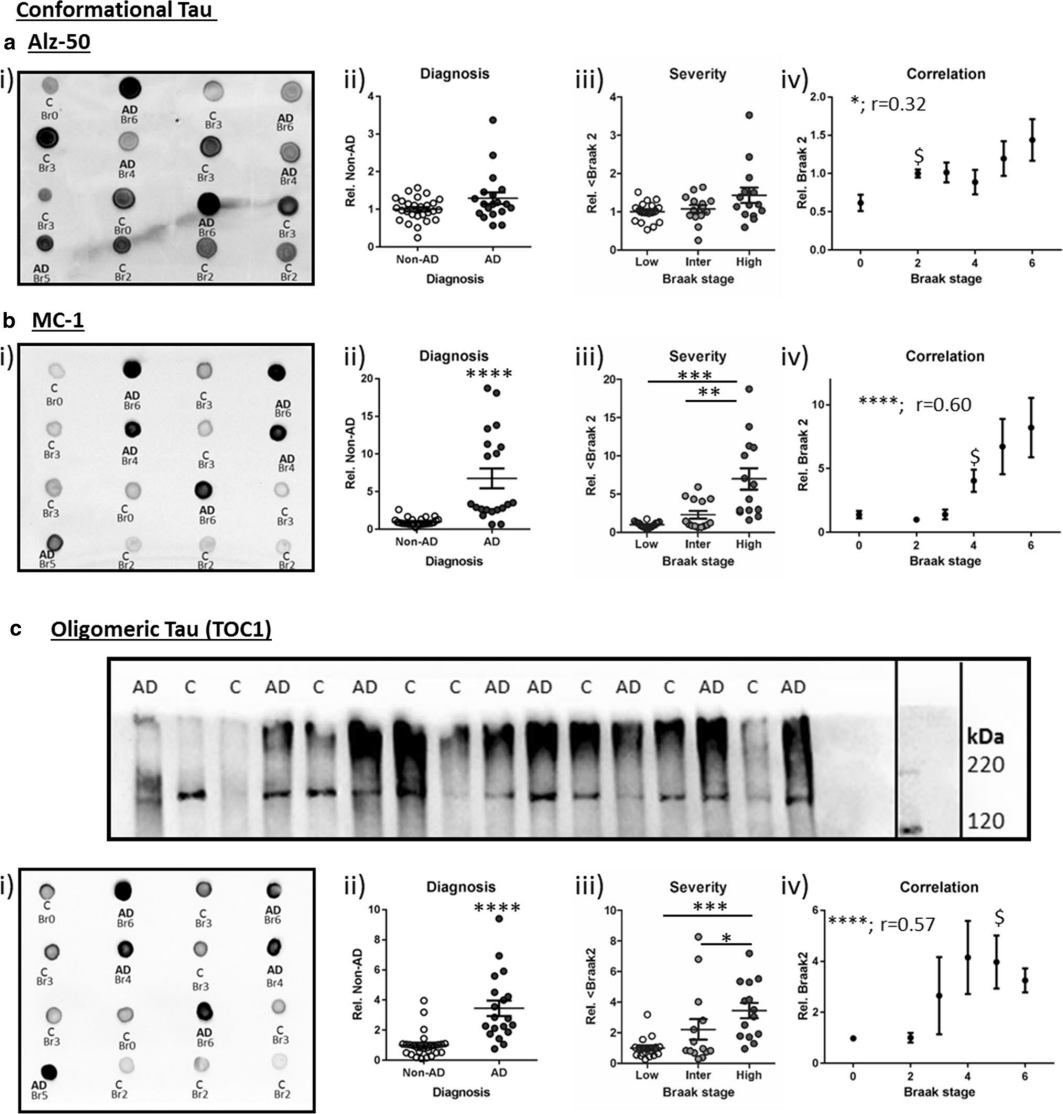
Conformational and oligomeric tau pathology. **a** Alz-50 and **b** MC-1 conformational tau and **c** oligomeric tau (tau-oligomeric-complex 1; TOC1)* dot blots*. The inset (*C*) illustrates an example Western blot for TOC1 run under non-reducing, non-denaturing conditions, labels show diagnosis status (*C* = non-AD and *AD*). In *A–C*, quantifications of immunoreactivity is categorised according to diagnosis (ii), severity (iii, low: Br 0–2, intermediate (Inter): Br 3–4 and High: Br 5–6) and correlation with Braak stage (iv). Significances are indicated as follows: *p < 0.05, **p < 0.01, ***p < 0.001, ****p < 0.0001 and Spearman’s correlation (*r*). The lowest Braak stage at which immunoreactivity was significantly elevated from Braak 0 is indicated by $

Alz-50 was the first antibody derived from paired helical filaments to recognise such a conformational change [37]. Here, its immunoreactivity was unaltered between Non-AD and AD cases (‘diagnosis’) and did not increase when considered across grouped Braak stages (Fig. 3ai–iii, *p* < 0.05). Despite a failure to detect differences between diagnosis and severity groups, a modest correlation between Alz-50 and Braak stage was observed (Fig. 3aiv, *r* = 0.32, *p* < 0.05), likely driven by the increase between individual stages (note Br 2 elevation compared to Br 0, *p* < 0.01). The use of the Alz-50 antibody may be confounded by its cross-reactivity with an uncharacterised developmentally regulated protein (Foetal Alz-50-reactive 1 clone protein, FAC1) [14], thus tau conformational changes were further probed with the related MC-1 antibody, which does not cross-react with FAC1 [37]. MC-1 immunoreactivity was distinct from that of Alz-50, detecting a highly selective signal in AD cf. Non-AD cases (Fig. 3bi + ii, *p* < 0.0001) and was enhanced in accordance with grouped Braak stages (Fig. 3biii, *p* < 0.0001), principally derived from the level of reactivity within the high category (low cf. high: *p* < 0.001; intermediate cf. high: *p* < 0.01). A strong correlation with MC-1 positive conformational tau and Braak stage was apparent (Fig. 3b, *r* = 0.60, *p* < 0.0001), and determined as elevated from ≥Br 4 relative to Br 0 (*p* < 0.05).

In addition to the emergence of conformational tau pathology, formation of oligomeric tau species has been proposed to correlate with behavioural deficits in animal models [7] and was also previously reported to be elevated in human AD cases [40, 64, 79]. TOC1 binds to a conformation-dependent epitope preferentially exposed upon oligomerisation (aa209–224) [79], and here recognised a single band when tested in Western blot applications under non-denaturing conditions (no DTT or boiling, ~180 kDa; Fig. 3c i, for full blot see Supplementary Figure 2). This band was previously established via SELDI-TOF MS as a tau dimer [63]. TOC1 immunoreactivity was characterised in all cases via native state dot blots (Fig. 3cii) and robustly increased for AD compared to non-AD cases (Fig. 3ciii, *p* < 0.0001), significantly tracked across grouped Braak stages (Fig. 3civ, *p* < 0.0001) and correlated with individual Braak stage (Fig. 3cv, *r* = 0.59, *p* < 0.0001). Relative to Braak stage 0 a significant elevation in reactivity emerged at Braak stage 5 (*p* < 0.05), although in a subset of cases a strong signal prior to this was apparent (Br 4 cf. Br 0, *p* = 0.07).

### Correlations of tau biomarkers

Correlative analysis of each pathological tau marker within our soluble preparation demonstrates a variable degree of agreement between all tau markers, with the exception of Alz-50 (Table 2). Interestingly, prominent markers for conformational and oligomeric tau yielded high correlations with differential phosphorylation epitopes (MC-1 with PHF-1 and TOC1 with CP13). No correlation with PMI, cortical pH or age was observed with any of the markers tested; this was further confirmed by ANCOVAs (all *p*’s > 0.05).

**Table 2:**
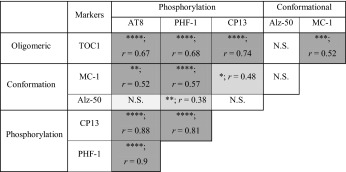
Correlations of tau pathology markers

Results are organised according to tau species subtypes and antibodies used

Significances (*p*) and outcome from Spearman’s correlation (*r*) between each marker and statistical reliability are shown and visualised by means of graded shading. * *p* < 0.05, ** *p* < 0.001, *** *p* < 0.001 and **** *p* < 0.0001. *N.S.* not significant

### APP processing and pathology

#### Amyloidogenic processing

**Fig. 4:**
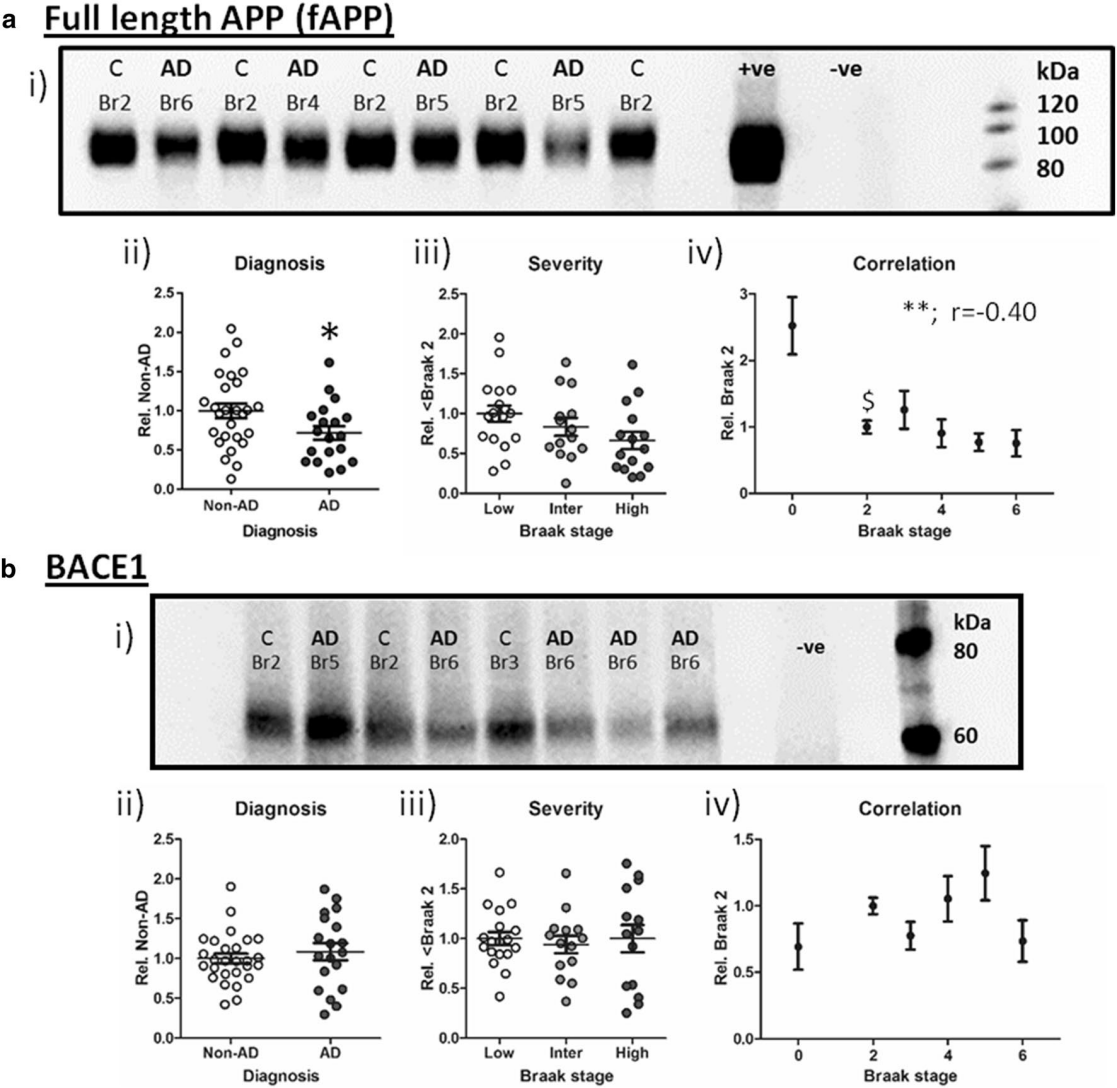
Amyloid precursor protein cleavage. Exemplary Western blots for **a** full-length APP detected via 6E10 immunoreactivity, and **b** β-secretase (BACE1) are illustrated. Braak stage (Br) and diagnosis (non-AD (*C*) cf. *AD*) are stated above each sample. Positive (+ve) control: human APP overexpressing mouse and negative (−ve) control (in **a**: wild-type C57/BL6 mouse and in **b**: BACE1^−/−^ mouse) are included. Total protein normalised immunoblot signals were analysed according to diagnosis (ii), severity (iii, low: Br 0–2, intermediate (Inter): Br 3–4 and high: Br 5–6) and Braak stage correlation (iv). Statistical results are presented as *p < 0.05, **p < 0.01 and Spearman’s correlation (*r*) are stated in the graphs. $ indicates the lowest Braak stage at which immunoreactivity differed significantly from Braak 0

Immunoblotting with the commonly used antibody 6E10, which is raised towards the human Aβ_1-16_ sequence, produced multiple bands in Western blots corresponding to various APP metabolites as well as full-length APP (fAPP). The major 6E10 immunoreactive band migrated between 80 and 120 kDa and was consistently observed in lysates from human samples and a hAPP overexpressing mouse (Fig. 4a i). This primary band equates to various post-translational modified species of fAPP and was evidently reduced between AD and non-AD cases (Fig. 4a ii, *p* < 0.05) but failed to reach significance across grouped Braak stages (Fig. 4a iii, *p* = 0.09). Levels of fAPP did, however, correlate (negatively) with the progression of Braak stage (Fig. 4a iv, *r* = −0.39, *p* < 0.01); post hoc analysis indicated this to be due to a significant reduction in the levels of fAPP as early as Braak stage 2 (*p* < 0.05 for Br 2 vs Br 0). Similar trends for the reduced expression of fAPP were also observed following the use of an N-terminal APP directed antibody (see Supplementary Figure 3).

The reduction of fAPP with diagnosis and disease severity may suggest a facilitation of APP cleavage via amyloidogenic secretases in AD cases. Several groups have previously reported a disease-dependent increase in BACE1 and thus a promotion of Aβ production pathways [24, 34]. However, despite the decrease of fAPP, BACE1 expression levels were unaltered in any of the analytical stratifications (Fig. 4b; *p* > 0.05 for all).

#### Aβ species

**Fig. 5:**
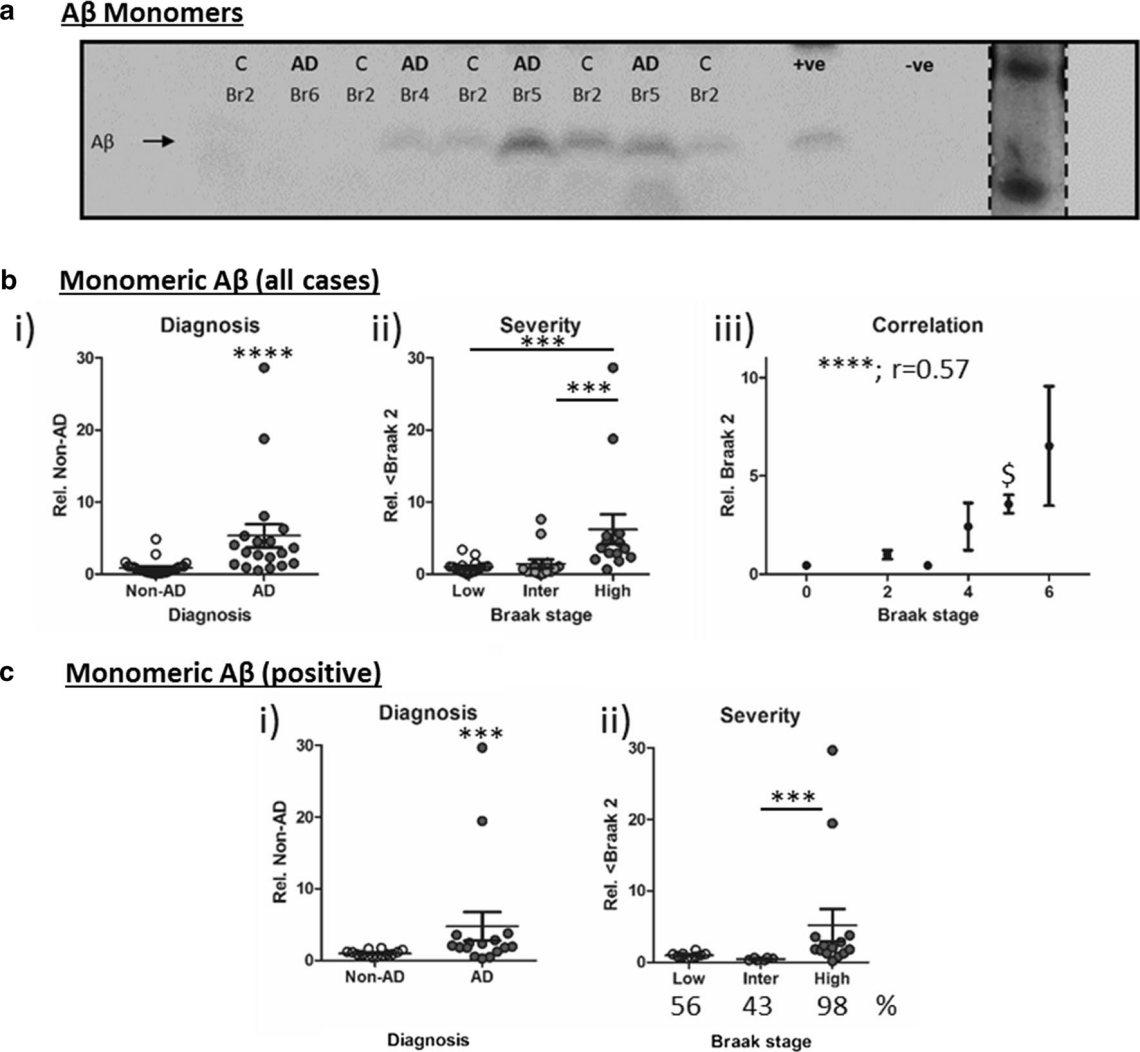
Monomeric β-amyloid (Aβ). Immunoblot for **a** monomeric Aβ (6E10 antibody). Diagnosis (Non-AD (*C*) cf. *AD*) and Braak stage is stated for each case. Positive (+ve) control: human APP overexpressing mouse and negative (−ve) control (wild-type mouse) samples are also shown. Size comparison was established via a Coomassie stained low molecular weight protein ladder (insert). Quantification was conducted either for all cases (**b**) or only for samples where a band was evident (**c**), the percentage (%) of cases in which monomeric Aβ was detected is stated below the graph. Total protein adjusted immunosignal stratified to i) diagnosis ii) severity (Low: Br 0–2, Intermediate (Inter): Br 3–4 and High: Br 5–6) and iii) correlation with individual Braak stage. Significances are illustrated as ***p < 0.001, ****p < 0.0001, Spearman’s correlation is also stated. $: indicates the earliest Braak stage at which immunoreactivity was higher than that observed at Braak stage 0

The detection of Aβ species, from monomers to oligomers, is problematic due to a number of confounding factors such as the cross-reactivity of many Aβ-directed antibodies with similar-sized non-Aβ APP metabolites and the potential for experimental parameters to modify Aβ self-oligomerization [80]. Probing standard Westerns blots with 6E10 detected immunoreactive bands migrating at 4 kDa equating to monomeric Aβ and 12 kDa band potentially equating to trimeric Aβ or C-terminal fragments of APP. The 12 kDa band was not quantified here, but also was detected in lysates from hAPP overexpressing mice, yet absent in wild-type mouse lysates (see example in Fig. 5a i). Initial quantification of monomeric Aβ (all cases), demonstrated a ~6-fold increase in monomeric Aβ levels in AD relative to non-AD cases (Fig. 5a ii, *p* < 0.0001) and an overall effect of grouped Braak stages (Fig. 5a iii, *p* < 0.001). Post hoc analysis demonstrated that significance was principally driven by the elevation at late stages (Br ≤ 2 cf. Br ≥ 5, *p* < 0.001 and Br 3–4 cf. Br ≥ 5, *p* < 0.001). Interestingly, a strong correlation with Braak stage was also established (*r* = 0.57, *p* < 0.0001) but the increased Aβ immunoreactivity did not reach significance from Braak stage 0 until stage 5 (*p* < 0.01).

**Fig. 6:**
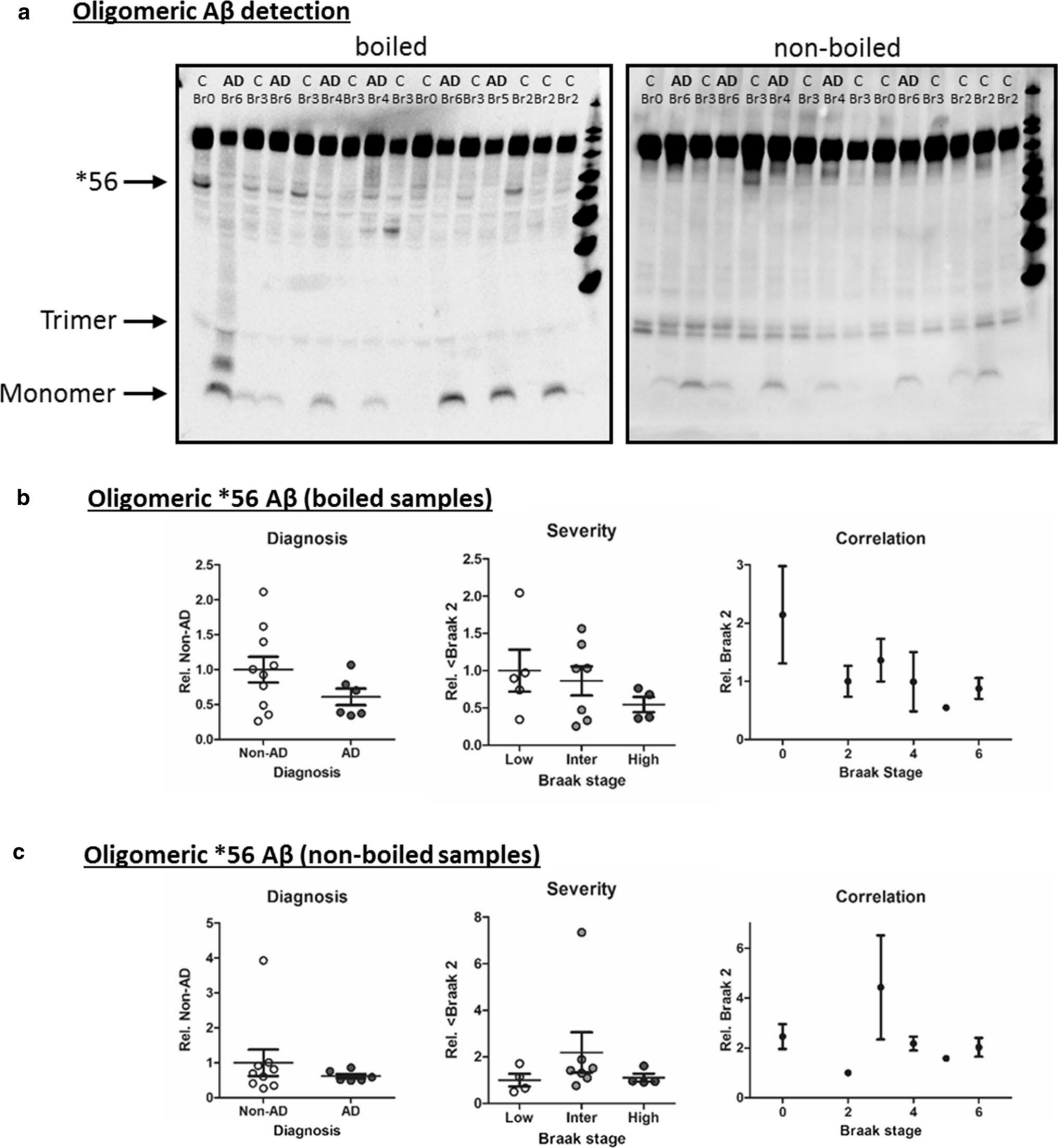
Heat-dependent detection of Aβ oligomers. **a** Side by side comparison of 6E10 immunoreactivty following boiling and non-boiling of samples prior to SDS-PAGE separation. Monomeric, trimeric and *56 Aβ bands are indicated alongside individual diagnosis (*C* non-AD control and *AD* cases) and Braak stage (Br) classifications. Quantification of *56 oligomeric Aβ levels according to i) diagnosis, ii) severity (Low: Br 0–2, intermediate (Inter): Br 3–4 and High: Br 5–6) and iii) correlation to individual Braak stage for boiled (**b**) and non-boiled (**c**) samples

Quantification of monomeric Aβ based on all cases is questionable as it relies on the inclusion of data from samples lacking a discernible band (i.e. signal value near background). The absence of detectable soluble Aβ in mildly dissociated tissues (manual homogenisation as opposed to sonification) using Western blots has previously been established [38], therefore, quantification in this fashion cannot be considered technically robust. Others have employed direct measurements using densitometry (for example [49, 67]), but failure to adjust for inter-blot differences in background and signal intensity may introduce additional noise into the data. More appropriate analysis arises from monomeric Aβ levels quantified only for those samples in which a band can be clearly detected (16 out of 19 AD samples, 14 out 27 non-AD samples). With this approach (termed monomeric Aβ positive, Fig. 5c i), monomeric Aβ was confirmed to be elevated for AD diagnosed cases relative to Non-AD controls (*p* < 0.01) and demonstrated a significant effect of grouped Braak staging (Fig. 5c ii *p* < 0.001), yet post hoc analysis demonstrated only a significant elevation between Braak stage 3–4 and 5–6 (*p* < 0.001) but not Braak stages 0–2 (*p* > 0.05). Normalisation of the data set to Braak stage 2 was not possible due to the inconsistent detection of monomeric Aβ within cases of low-stage pathology. Other higher molecular Aβ aggregates have attracted attention in the past, for example, the frequently reported dodecameric Aβ*56 [48, 49]. A corresponding band was detected here following longer exposure times, which revealed several additional bands on Western blots probed with 6E10 (Fig. 6a). In a subset of cases (*n* = 16), we attempted to quantify and validate the *56 band. Although no significant difference between diagnosis or severity groups could be established (Fig. 6b i–ii), an apparent decline in *56 levels with Braak stage was observed (Fig. 6b iii) in agreement with previous reports [49]. However, Western blot protocols pose several technical issues for the identification of Aβ oligomers due to species modifications induced by reducing agents and heating of samples. Therefore, samples were also run under quasi non-denaturing conditions as conducted for TOC1. Under these near-native state conditions (Fig. 6a), the detection of *56 was greatly diminished and no consistent alteration was observed in any analytical stratification for any group (Fig. 6a + c), indicative that potential artefacts induced by standard SDS-PAGE methods had likely modified native Aβ species.

**Fig. 7:**
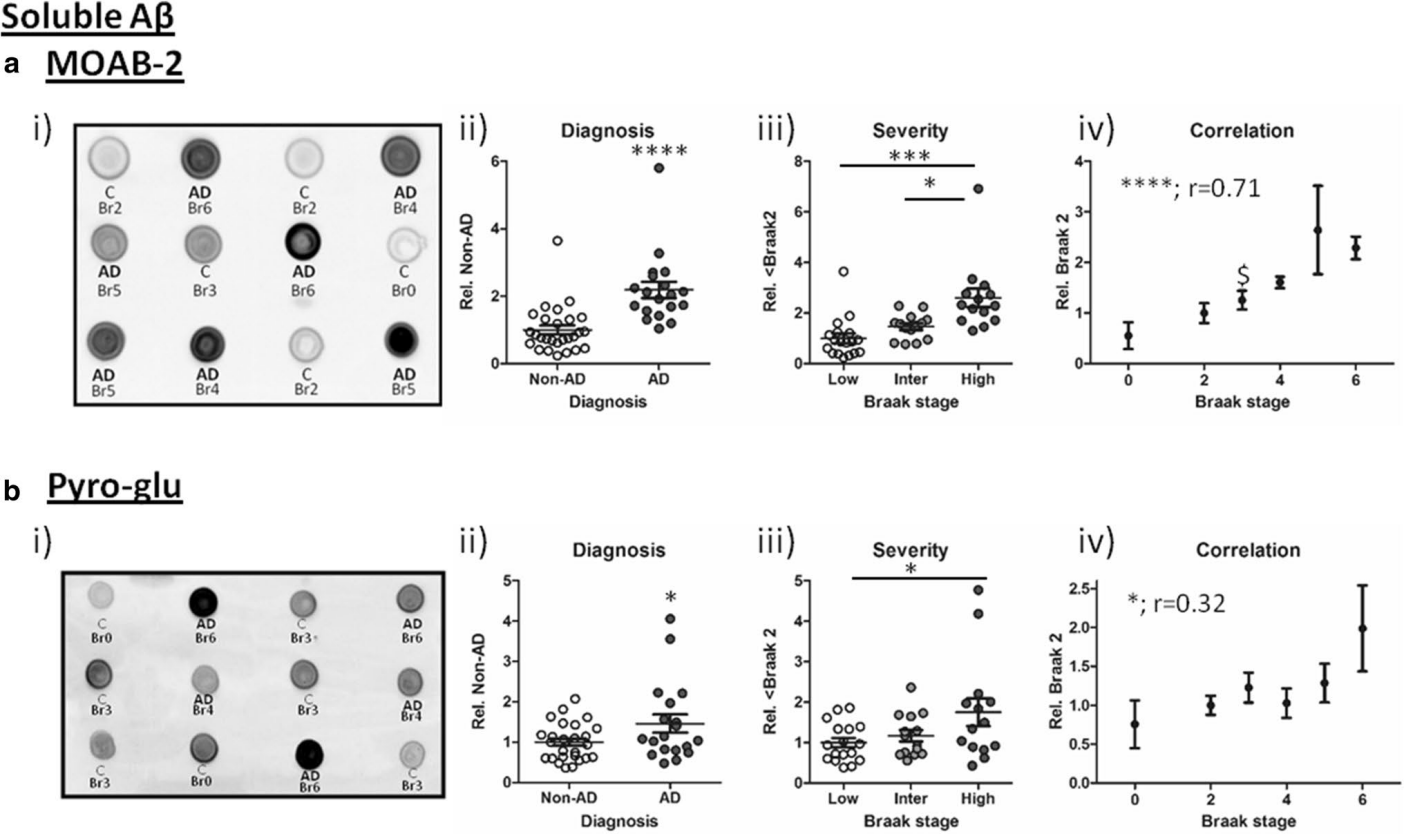
Soluble Aβ detection. Example immunoblots for **a** i) MOAB-2 and **b** i) pyro-glu reactive Aβ; each case is labelled with corresponding diagnosis [Non-AD (*C*) cf. *AD*] and Braak stage. Quantified signals normalised to total protein are shown stratified to ii) diagnosis iii), severity (Low: Br 0–2, Intermediate (Inter): Br 3–4 and High: Br 5–6) and iv) correlation with Braak stage. *p < 0.05, ***p < 0.001, ****p < 0.0001 and Spearman’s correlation* r* is stated in iv. $ denotes lowest Braak stage at which immunoreactivity differed from Braak 0

As an alternative means of quantifying soluble Aβ load, non-denaturing dot blots were stained with the Aβ-selective antibody MOAB-2, which does not cross-react with APP or other metabolites [77]. Here, Aβ was consistently detected in all AD cases, with levels robustly elevated in diagnosed relative to Non-AD samples (Fig. 7a ii, *p* < 0.0001); the signal increased progressively across grouped Braak stages (Fig. 7a iii, *p* < 0.0001). Further statistical comparison revealed strongest elevations in Braak 5–6 cases compared to Braak 0–2 (*p* < 0.001) and Braak 3–4 (*p* < 0.05). The progressive increase in soluble Aβ load was further confirmed by the positive near-linear correlation of MOAB-2 reactive species with Braak stage (Fig. 7a iii, *r* = 0.71, *p* = 0.0001). MOAB-2 levels were initially detected as enhanced at Braak stage 3 relative to Braak stage 0 (*p* < 0.05).

Next, cases were further probed for post-translational modification of Aβ species with pyro-glu [21]. In Western blots, a single immunoreactive band was identified migrating ~12 kDa, which in a subset of samples approached significant elevations based on diagnosis and when samples grouped in low, intermediate and high Braak stages (*p* = 0.07 and *p* = 0.08, respectively), but did correlate with individual Braak stages (*r* = 0.32, *p* < 0.05; data not shown). When all cases were characterised in non-denaturing immuno-dot blot conditions, pyro-glu Aβ immunoreactivity was enhanced based on diagnosis criteria (Fig. 7b i, *p* < 0.05), increased in line with grouped Braak stages (Fig. 7b ii, *p* < 0.05) and correlated with individual Braak stage (Fig. 7b iii, *r* = 0.32, *p* < 0.05). Post hoc analysis indicated that significance was largely driven by late-stage pathological changes (Br 5–6 cf. Br ≤ 2, *p* < 0.05), yet no individual Braak stage was significantly elevated from Br 0 cases. As can be seen in scatter plots in Fig. 7b, significance was primarily driven by two highly reactive AD cases, however, further investigation (Grubb’s outlier test) did not report these as significant (*p* > 0.05).

**Fig. 8:**
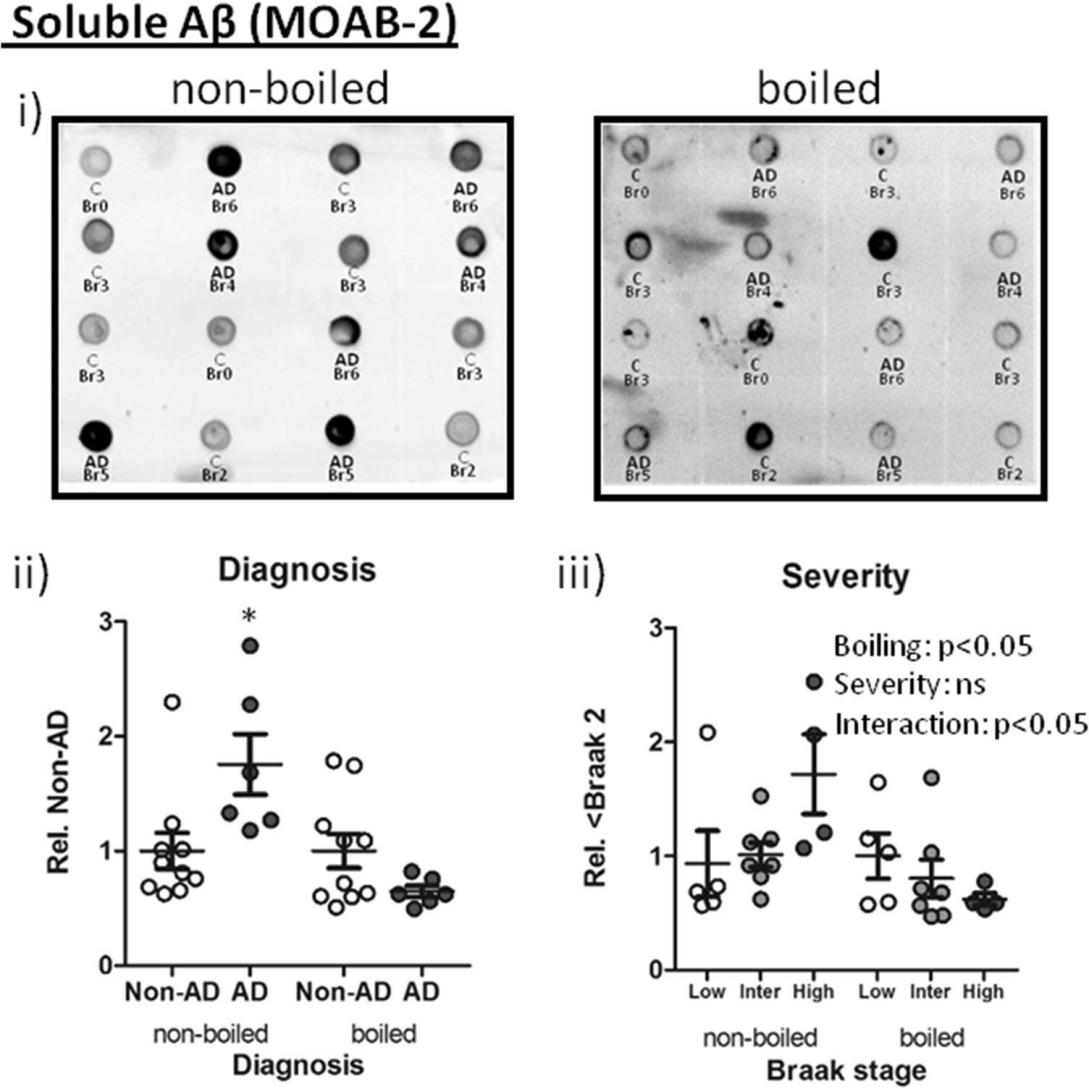
Heat sensitivity of soluble Aβ (MOAB-2 epitope). (i) Dot blots of either boiled or non-boiled samples probed with MOAB-2 for oligomeric Aβ. Diagnosis [non-AD (*C*) and AD] and Braak stage (Br) are shown for each sample. Matched samples for boiled vs non-boiled conditions were analysed according to diagnosis (ii) and severity (low: Br 0–2, intermediate (Inter): Br 3–4 and high: Br 5–6) (iii). Statistical outcome of a two-way ANOVA is reported in the corresponding graph. *p < 0.05 and ***p < 0.001

To further characterise the impact of heating on Aβ and their detection, boiled and unboiled lysates were transferred onto nitrocellulose membranes. Under boiling conditions, the diagnosis-specific increase of Aβ as detected by MOAB-2 was abolished and even appeared reduced relative to Non-AD cases (Fig. 8a i–ii, *p* = 0.09). The heat-sensitive nature of MOAB-2 mediated Aβ detection was further confirmed by a two-way ANOVA, comparing across grouped Braak stages (Fig. 8a iii, Braak stages: *F*_(2,26)_ = 0.83, p > 0.05, boiling: *F*_(1,26)_ = 5.9, *p* < 0.01 and interaction: *F*_(2,1)_ = 3.8, *p* < 0.05). In comparison, the detection of phospho-Tau was unaffected by boiling (see Supplementary Figure 4).

#### Correlation between Aβ-related biomarkers

The various Aβ-related markers failed to demonstrate a similar interrelated pattern as observed for tau (Table 3). Nevertheless, fAPP measurements inversely correlated with quantified levels of MOAB-2 reactive Aβ, which itself correlated with monomeric and pyro-glu Aβ, as one would expect for a signal derived from a metabolite of APP. No correlation or effect of cortical pH or PMI as covariates was observed for any of the Aβ markers examined here (*p* > 0.05).

**Table 3:**
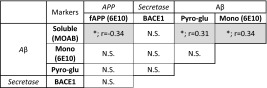
Correlations between amyloid pathology markers

Components of the amyloidogenic cascade listed are full-length APP (fAPP) as detected via 6E10 APP antibody, β-secretase (BACE1), monomeric Aβ (mono Aβ) as detected by 6E10, soluble Aβ as detected by MOAB-2 and pyro-glu Aβ

Significances (*p*) and Spearman’s correlation (*r*) between each component are provided. * *p* < 0.05. *N.S.* not significant

#### Tau and Aβ: mutual correlations

The correlations identified for tau and amyloid markers imply a relationship of components within the categories of tau and amyloid species. Determining correlations between these pathologies is of critical importance, particularly in light of the early stage emergence of both soluble tau and amyloid (Table 4). Accordingly, soluble MOAB-2 reactive Aβ was the only Aβ-related marker to correlate with all markers of tau pathology, and best matched with phospho-tau markers PHF-1 and AT8 (*r* > 0.63, *p* < 0.0001 for both). These observations were further strengthened by the reciprocal, inverse correlations of oligomeric (TOC1), conformational (MC-1) and phospho-tau (CP13 and AT8) with total fAPP. In contrast to soluble MOAB-2-reactive Aβ, monomeric Aβ and pyro-glu Aβ correlated more selectively with phospho-tau and oligomeric tau epitopes.

**Table 4:**
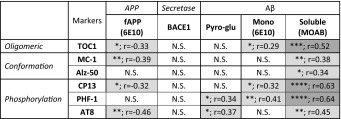
Correlations between tau and amyloid markers

Data are organised according to the components of the amyloidogenic cascade (horizontal axis) or tau pathology (vertical axis). Spearman’s correlation (*r*) between each component and statistical strength (*p*) is shown. Intensity of shading visualises strength of correlation

* *p* < 0.05, ** *p* < 0.001, *** *p* < 0.001 and ****  *p* < 0.0001. *N.S.* not significant

#### Pathological correlates of Braak staging and cognition

The overall robust correlations of both tau and Aβ markers with Braak stages are summarised in Table 5 and listed alongside associations obtained with cognitive measures. Outcomes were in close agreement with each other, reporting strong correlations with all phospho-tau markers but also with conformational tau (MC-1) and oligomeric tau (TOC1). Critically, for amyloidogenic processing soluble MOAB-2 reactive Aβ consistently reported a high level of correlation with cognitive decline, approximately matching the correlative strength of tau markers. Monomeric Aβ also strongly correlated with cognitive decline, however, this must be viewed with caution due to the lack of reliable detection in some AD cases.

**Table 5:**
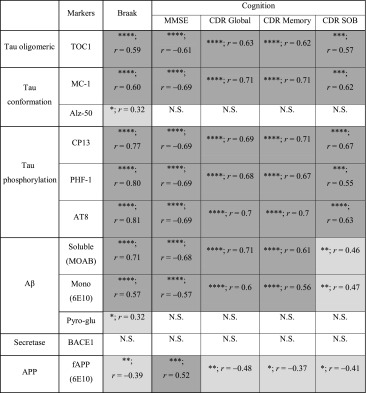
Correlation of tau and amyloid markers with Braak stage and cognitive scores

Significances (*p*) and Spearman’s r correlations are given for each marker (for details, see previous Tables) with Braak stage as well as with cognitive scores [established via the Mini mental state exam (MMSE) and Global, Memory and Sum of Box (SOB) variants of the clinical dementia rating (CDR)]. The strength of correlation is indicated by the intensity of shading. * *p* < 0.05, ** *p* < 0.01, *** *p* < 0.001 and **** *p* < 0.0001, *N.S.* not significant

## Discussion

### Taupathology

Quantification of soluble, abnormally phosphorylated tau detected in brain lysates was highly predictive of the clinical diagnosis and rose with the severity of neuropathological classifications, regardless of the phospho-epitope. In agreement with the stereotypical progression of NFTs over the course of AD, phosphorylation at the AT8 epitope was first significantly elevated at Braak stage 4 [2, 15]. Classically based on the detection of NT and NFTs via argyrophilic stains, modern Braak staging utilises AT8 and thus includes the additional detection of pre-tangle tau. The accumulation of soluble, phosphorylated tau is evident within the temporal cortex and precedes mature tau lesions in many low Braak stage (I–III) cases [2]. Here, tau species detected in our soluble fraction most likely reflect changes within pre-tangle neurons rather than NF and NFTs, and already appeared at Braak stage 2 (CP13). All three phospho-tau antibodies strongly correlated with Braak stages, in close agreement with previous reports [84]. This was nevertheless surprising given the well-documented post-mortem reduction in phosphorylation [23], largely due to the energy-independent activity of phosphatases. Although samples used in the current study had longer PMIs compared to those from some previous publications, no impact of PMI as covariate or correlation of any marker with PMI was observed. Moreover, no evidence for protein degradation was detected in either blotting techniques. Ultimately, the long PMIs may have resulted in an under-estimation of pathological tau phosphorylation but strong correlations of phospho-tau markers with other pathological markers as well as cognition provide evidence for reliable disease tracking.

The rise of tau phosphorylation at Braak stage 2 was mirrored by the conformation-specific antibody Alz-50, although as previously discussed non-specific FAC1 binding may be a confounding factor here [14]. The improved MC-1 antibody [37, 81] demonstrated a superior signal associated with diagnosis, Braak staging and cognitive decline. However, MC-1 reactive tau was not elevated significantly until Braak stage 4, at which point both CP13 and AT8 phosphorylation markers were also raised. Tau’s ability to adopt folded MC-1-reactive conformation may be increased in situ following phosphorylation at multiple sites as has been observed in vitro [36]. Hence, changes in phosphorylation may indeed be required before conformational changes can occur [51].

Previous studies have shown a robust increase in TOC1 reactivity in AD compared to control samples [40, 63], but have not assessed the association of TOC1 with disease progression and cognitive impairment scores. Thus, an important conclusion from our data is that oligomeric tau appears to be a strong predictor of cognitive scores, clinical diagnosis and neuropathological severity. Recent experimental studies have indeed linked low-weight oligomeric tau rather than fibrillary species to e.g. increased neuronal toxicity, impaired axonal transport, inhibition of synaptic plasticity, synaptic loss, mitochondrial impairment and memory deficits [22, 46, 47, 63, 64]. Overall, our data lend direct support to the patho-physiological relevance of non-filamentous, oligomeric tau. Nevertheless, strong correlations with cognitive decline were also observed for conformational and phospho-tau as previously reported in humans [25, 30] and animal models [43, 44, 83]. Our data expand on the suggested disruptive role of monomeric phospho-tau [41], and suggest oligomerization caused by pathological modification of monomeric tau to play a key role. Factors causing the formation of oligomeric tau species remain largely undefined, but native tau does not appear to readily self-aggregate under near-physiological conditions [20, 42]. Not surprisingly, several post-translational modifications facilitate tau aggregation, and our current work implies facilitation of oligomerisation via multiple phospho-epitopes, consistent with the extensive co-localization between TOC1 and phospho-ser422 tau, an early pre-tangle phospho-epitope [28], in the EC and cholinergic basal forebrain [63]. Conversely, tau oligomers do not readily co-localise with markers of more mature fibrillary tau species [63, 79]. Taken together, the coincident appearance of phospho-epitopes and oligomerization is supportive of the suggestion that abnormal phosphorylation may facilitate tau oligomerization [35] thus generating pathological tau entities early in the disease process. Nevertheless, future studies are required to better define which phospho-epitopes are directly involved in this process in situ.

With respect to measures of cognitive ability, all markers except Alz-50 provided robust associations. As we focused here on tau species that are soluble in mild detergent, non-denaturing conditions, our findings strongly suggest that soluble tau is sufficient and relevant for cognitive impairment, and adds support to the proposition that soluble pathological species are more relevant to disease progression as opposed to the NFT and plaque inclusions [11]. Correlations obtained here were clearly stronger than previously reported for NT and NFTs in AD and MCI cases [59], despite the limited number of cases and varying origin.

### APP processing and soluble Aβ species

Early stage alterations in Aβ markers were twofold: a diagnosis-specific, progressive decline in fAPP and a corresponding increase in MOAB-2 reactive Aβ species. The progressive decline of fAPP is in contrast to many other studies, which found either no change [8, 60] or an increase [3, 82]. There are several possible explanations for this inconsistency, but perhaps the most pertinent is the composition of sample groups. None of the previous investigations had quantified APP levels according to individual Braak staging, and here the greatest loss of APP was seen between Braak stage 0 and 2, two stages often pooled into a non-AD control groups. Thus, the early loss of total APP levels may have been overlooked.

The observed reduction in APP may be a consequence of transcriptional downregulation [75], however, the strong negative correlation between total APP and soluble Aβ species suggests increased amyloidogenic APP cleavage. Whilst no change in the expression levels of BACE1 was observed (in contrast to the investigations of others [24, 34]), we cannot rule out the possibility of increased BACE1 activity in AD [1, 24, 72].

Regarding Aβ species, monomeric, pyro-glu modified and total soluble levels were elevated according to diagnosis and Braak stage severity. These forms of Aβ also robustly correlated with individual Braak stages and both monomeric and soluble levels of Aβ further correlated, albeit to varying degrees, with measurements of cognition. Our data also suggest that detection of monomeric Aβ via Western blots is not always possible, particularly in non-AD cases, but also in a number of AD cases. The apparently low levels of soluble monomeric Aβ agree with recent findings which indicate a potential over-estimation of respective Aβ species following aggressive tissue homogenisation via sonification [38]. Genuine soluble, monomeric Aβ species may in some cases be below the detection limit of conventional Western blotting, even in cases with Braak stage 5 pathology [38]. Detection of Aβ may be further complicated, as even common antibodies such as 6E10 and 4G8 may only bind sub-species of Aβ, dependent on conformation [33]. Regardless of the analytical parameters, monomeric Aβ was elevated in agreement with both diagnosis and Braak stage, closely matching data from others [67]. Yet, a significant increase occurred only in advanced pathological cases and did not follow a progressive scale, in agreement with previous observations [49].

### Oligomeric Aβ

Our data strongly suggest that chemical and physical parameters of standard Western Blot techniques are not suitable for the reliable detection of native Aβ species. Although numerous potential SDS-stable Aβ oligomers were visible following the overexposure of 6E10 blots, it is far from clear if any of these entities are native to the brain and/or disease relevant. Specifically, this relates to the oligomeric species known as *56, a dodecamer of Aβ, which has been identified in several animal models [18] including our own [65], and found to decrease as a function of disease [49]. It has been suggested to be an indicator of a prodromal time window, triggering degenerative cascades before sequestration into increasingly insoluble plaque deposits. Here, such an inverse relationship between disease severity and this oligomeric Aβ species also seemed apparent, declining from early Braak stage cases to high pathology cases. However, the utility of such SDS-stable oligomeric species must be questioned when considering that the presence of SDS, even at low concentrations (0.2 %) can greatly enhanced the abundance of dimeric [80] trimeric [10] and higher, multimeric Aβ species [5, 31, 32, 66]. Furthermore, and in line with the impact of heat and reducing agents such DTT to modulate amyloidogenic β-sheet aggregation [13, 45, 69], we demonstrate that the relative abundance of *56 was strongly reduced and any correlation with disease progression lost in the absence of such treatments. This suggests that some of the previous data may be due to technical artefacts. The observation of highest levels of *56 in Braak stage 0 cases is also somewhat at odds with the hypothesis of *56 being pathologically causative, as one would anticipate lowest levels in healthy adults and thus at least an initial elevation between what is considered a pathology-free status (Br 0) and those cases assumed to be at risk of developing AD (Br 2 and 3).

Contrary to SDS-PAGE resolved oligomers and monomers, dot blot detection of soluble unmodified oligomeric Aβ allowed consistent detection and demonstrated a robust disease-specific increase and a progressive correlation with Braak stages and cognitive decline. Critically, this relationship was completely abolished following heating and induced a similar inverse relationship with disease progression, as observed for *56. Together, our data strongly suggest that artificial modification of Aβ species has hampered many previous studies, via promotion of soluble Aβ species into oligomeric configurations which may mask epitopes or destroy binding sites. The heat-sensitivity likely explains the reported failure of MOAB-2 immunoreactivity to detect AD cases from pre-heated basal forebrain lysate following dot blot analysis by others [4] and thus highlights the need to carefully review both sample preparation and subsequent methods for Aβ quantification.

Even though our approach does not allow the detection of particular Aβ species, MOAB-2 does not cross-react with APP or other metabolites, and it binds a C-terminal epitope with stronger affinity to more toxic Aβ_1–42_ than to Aβ_1–40_, independent of aggregation state [77]). The increase in total soluble levels of Aβ with disease diagnosis is in line with ELISA-based measurements from cortical tissue [19, 50]. Compared to measurements of insoluble Aβ [58] and plaque load [6, 59, 62], MOAB-2 reactivity provides much better correlation with cognitive decline (ranging from 0.46 up to 0.71) as well as improved correlation with disease progression as established with the biochemical staging of Aβ [67]. Similarly, the immunoreactive levels of soluble Aβ correlated with all tau markers tested (see below). In comparison, strong associations were not observed for pyro-glu Aβ: Although selective for diagnosis, elevations were only apparent between low and high Braak stage cases. Furthermore, pyro-glu Aβ only weakly correlated with individual Braak stages and failed to correlate with any measure of cognition. This is largely at odds with previous reports of disease specificity [52] and toxicity [61], although in agreement with previous work where a correlation was established between levels of pyro-glu-modified Aβ and tau phosphorylation [52].

### Early co-localisation of soluble tau and amyloid pathology

It is fairly well established that Aβ plaques precede the emergence of NFTs in the neocortex, while NFTs are formed earlier in limbic areas [74]. However, our findings clearly indicate that initial emergence of tau pathology coincides with increased soluble Aβ in Brodmann area 21 as early as Braak stages 2–3. This pathology therefore precedes the emergence of mature NFTs, classically determined at Braak stage 4 in this brain region. Thus, our data suggest a closer overlap for soluble Aβ and tau species in the temporal lobe, contrasting with the spatial separation of the two toxic pathways based on the mature fibrillary forms of these proteins. Thus, at least in Brodmann area 21, the early co-localization of soluble tau and Aβ pathology implies a close spatiotemporal relationship between these two pathologies.

In summary, our findings are supportive of the coincident emergence of disease-relevant, soluble, pre-fibrillar forms of both Aβ and tau during the earliest stages of AD. Potentially toxic protein species identified here strongly correlate with Braak staging and cognitive decline. Causal and functional links between Aβ and tau pathways will remain a hotly debated topic, but here we provide evidence that both pre-fibrillar Aβ and tau appear as likely contributors to the pathogenesis of AD. Late-stage insoluble protein aggregates may have very little relevance to the pathogenic mechanisms of disease, and therefore targeting pre-fibrillar tau and/or Aβ species will be a more effective therapeutic strategy for AD.

## Electronic supplementary material

Below is the link to the electronic supplementary material.

Supplementary material 1 (TIFF 3198 kb)

Supplementary material 2 (TIFF 1152 kb)

Supplementary material 3 (TIFF 1545 kb)

Supplementary material 4 (TIFF 1582 kb)

Supplementary material 5 (DOCX 25 kb)
