# Supplementary figures and images for: Soluble pre-fibrillar tau and β-amyloid species emerge in early human Alzheimer’s disease and track disease progression and cognitive decline
Source: Acta Neuropathol. 2016 Oct 21;132(6):875–95. doi: 10.1007/s00401-016-1632-3 (PMC5106509; doi:10.1007/s00401-016-1632-3)

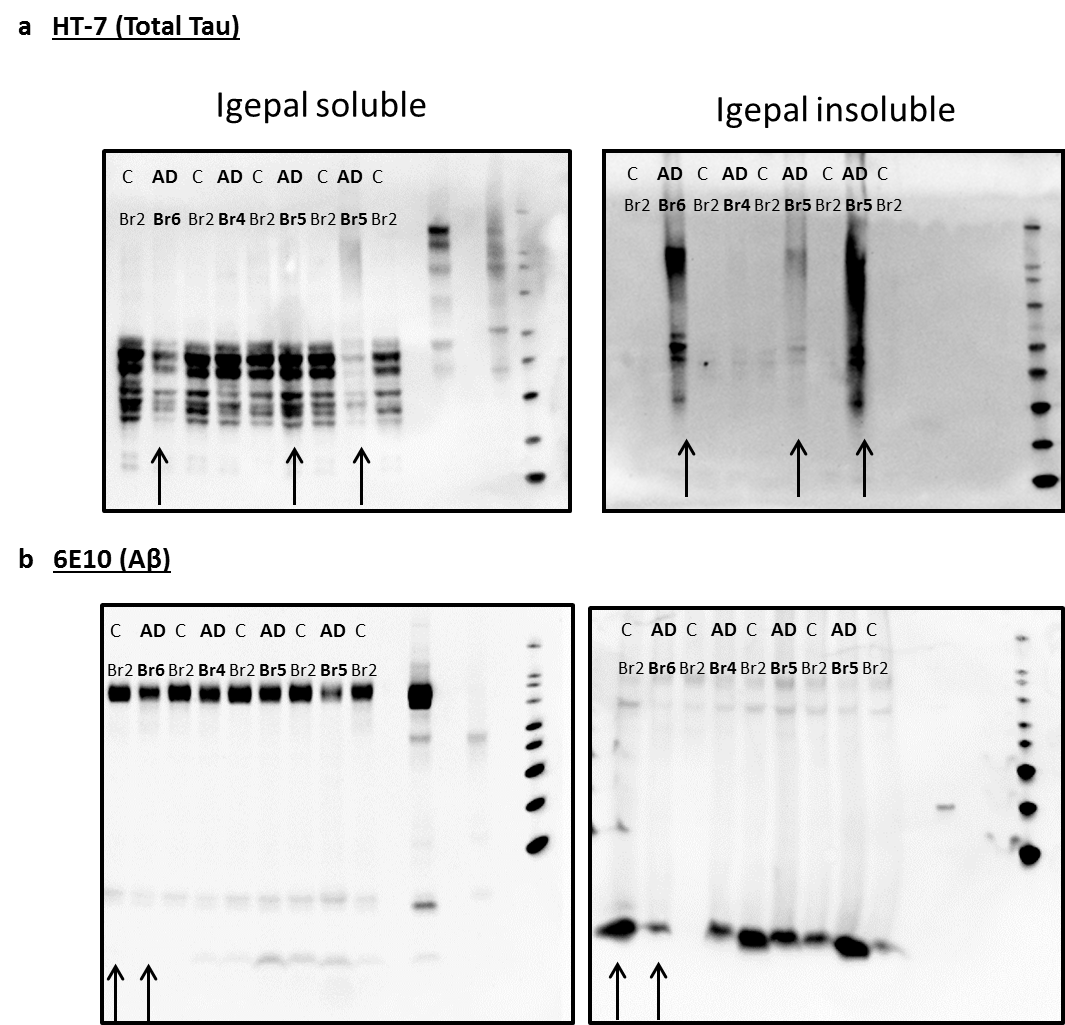

Supplement: Supplementary file 1 — Supplementary material 1 (TIFF 3198 kb) [file 401_2016_1632_MOESM1_ESM.tif]

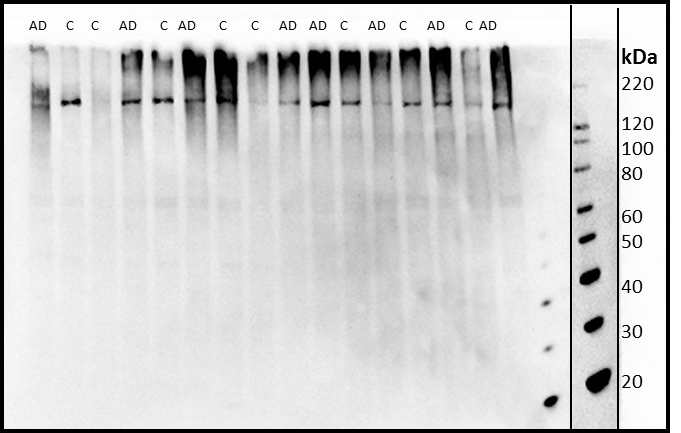

Supplement: Supplementary file 2 — Supplementary material 2 (TIFF 1152 kb) [file 401_2016_1632_MOESM2_ESM.tif]

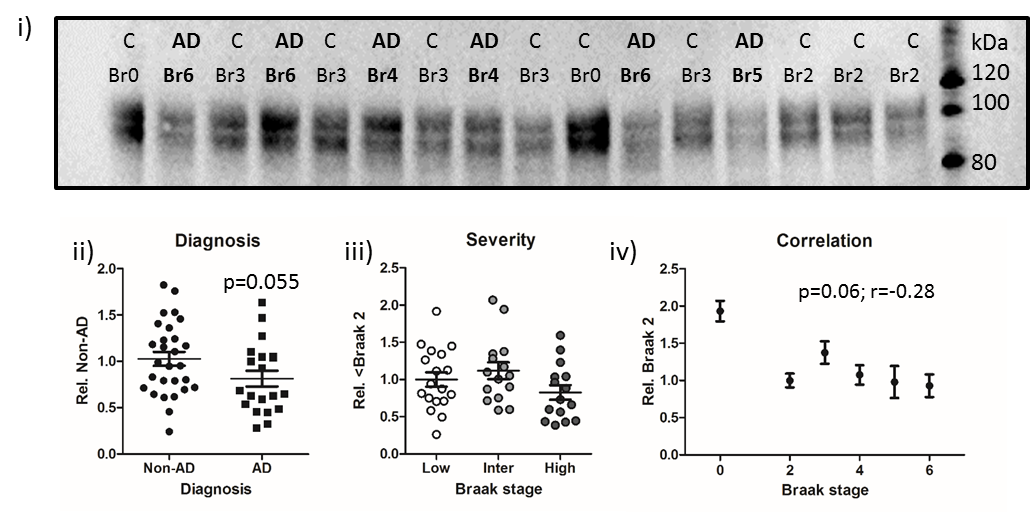

Supplement: Supplementary file 3 — Supplementary material 3 (TIFF 1545 kb) [file 401_2016_1632_MOESM3_ESM.tif]

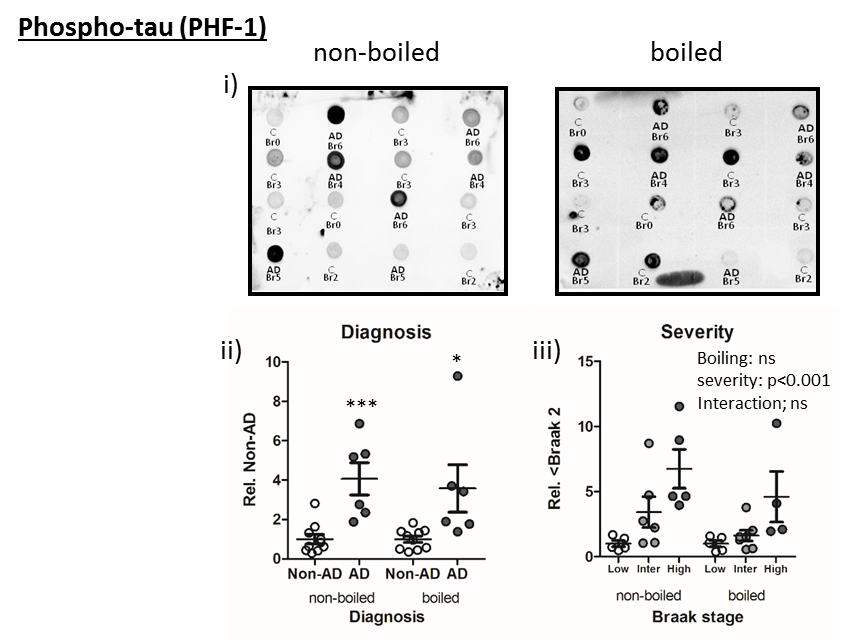

Supplement: Supplementary file 4 — Supplementary material 4 (TIFF 1582 kb) [file 401_2016_1632_MOESM4_ESM.tif]
