# Supplementary material for: Soluble pre-fibrillar tau and β-amyloid species emerge in early human Alzheimer’s disease and track disease progression and cognitive decline
Source: Acta Neuropathol. 2016 Oct 21;132(6):875–95. doi: 10.1007/s00401-016-1632-3 (PMC5106509; doi:10.1007/s00401-016-1632-3)
Supplement: Supplementary file 5 — Supplementary material 5 (DOCX 25 kb) [file 401_2016_1632_MOESM5_ESM.docx]

| Case | Gender | Age | Braak | CERAD | Neuropathological diagnosis / observations | PMI | pH |
| --- | --- | --- | --- | --- | --- | --- | --- |
| 1 | M | 74 | 0 | - | Control | 23 | 6 |
| 2 | M | 78 | 0 | - | Control | 11 | 6.1 |
| 3 | M | 78 | 0 | C0 | Control | 56 | - |
| 4 | M | 96 | 2 | C0 | Control | 21 | - |
| 5 | F | 84 | 2 | C0 | Control, normal aged brain. | 72 | 6.6 |
| 6 | F | 84 | 2 | C0 | Control, normal aged brain. | 30 | 6.14 |
| 7 | F | 79 | 2 | C0 | Control, rare microscopic foci of metastatic tumour. Otherwise normal aged brain. | 18 | - |
| 8 | M | 85 | 2 | C0 | Control, massive brainstem haemorrhage. | 24 | 5.96 |
| 9 | F | 89 | 2 | C1 | Control, normal ageing. | 12 | 6.48 |
| 10 | F | 90 | 2 | - | Control | 44 | 6.1 |
| 11 | F | 90 | 2 | - | Control case. TDP-43 pathology in hippocampus and amygdala | 87 | 6.7 |
| 12 | M | 78 | 2 | C0 | Control | 51.5 | - |
| 13 | F | 94 | 2 | C0 | Control | 29.5 | - |
| 14 | F | 103 | 2 | C1 | Control, Alzheimer pathology/normal ageing TDP43opathy | 41 | - |
| 15 | M | 74 | 2 | C1 | Control, normal ageing changes | 92 | - |
| 16 | F | 91 | 2 | C1 | Normal ageing | 78 | 6.47 |
| 17 | M | 80 | 2 | C0 | Cognitively normal control | 16 | 6.36 |
| 18 | M | 88 | 2 | C0 | Cognitively normal control | 26 | 6.1 |
| 19 | F | 89 | 3 | - | Control, disseminated metastatic poorly differentiated neuroendocrine carcinoma | 56 | 6.9 |
| 20 | F | 84 | 3 | - | Control, early Alzheimer type changes (predominant Tau pathology) | 89 | 6.6 |
| 21 | F | 87 | 3 | - | Control, mild Alzheimer-type pathology | 67 | 6 |
| 22 | M | 95 | 3 | C2 | Control, mild AD changes | 26 | - |
| 23 | F | 85 | 3 | C0 | Control | 13.5 | - |
| 24 | F | 86 | 3 | C2 | Control, early/mild Alzheimer's disease | 36 | - |
| 25 | M | 78 | 3 | C2 | Control, mild Alzheimer's pathology | 42 | - |
| 26 | F | 92 | 3 | C2 | Control, early Alzheimer's disease / normal ageing | 101 | - |
| 27 | M | 92 | 3 | C0 | Control, AD pathology in limbic stage (age associated/ control) | 50 | 5.4 |
| 28 | F | 88 | 4 | C2 | Alzheimer's disease | 33 | 6.8 |
| 29 | M | 80 | 4 | - | Alzheimer's disease /extensive amyloid angiopathy | 51 | 6.1 |
| 30 | F | 88 | 4 | C2 | Alzheimer’s disease. | 44 | 6.01 |
| 31 | M | 77 | 4 | C2 | Alzheimer's disease | 87 | 6.05 |
| 32 | F | 81 | 4 | C3 | Alzheimer's disease | 26 | - |
| 33 | M | 85 | 5 | C3 | Alzheimer’s disease. | 24 | 6.09 |
| 34 | M | 82 | 5 | C3 | Alzheimer’s disease / tau-positive astrogliopathy. | 72 | 6.15 |
| 35 | M | 88 | 5 | C1 | Alzheimer's disease | 66 | 6.6 |
| 36 | M | 88 | 5 | C3 | Alzheimer's disease | 78 | 6.2 |
| 37 | F | 82 | 5 | C3 | Alzheimer's disease | 22 | - |
| 38 | M | 82 | 5 | C3 | Alzheimer's disease / amyloid angiopahty | 41 | 6.36 |
| 39 | M | 79 | 6 | C3 | Alzheimer's disease / moderate amyloid angiopathy | 24 | 6.9 |
| 40 | M | 71 | 6 | C3 | Alzheimer's disease /extensive amyloid angiopathy | 20 | 6.3 |
| 41 | M | 79 | 6 | C3 | Alzheimer's disease / moderate amyloid angiopathy | 38 | 6.3 |
| 42 | M | 90 | 6 | C3 | Alzheimer's disease | 69 | 6.5 |
| 43 | F | 89 | 6 | C3 | Alzheimer's disease | 22 | - |
| 44 | F | 87 | 6 | C3 | Alzheimer's disease | 51 | - |
| 45 | F | 89 | 6 | C3 | Alzheimer's disease | 65 | - |
| 46 | F | 84 | 6 | C3 | Alzheimer's disease | 52 | - |

**Supplemental Table 1. Neuropathological information for Individual cases**. Cases are organised according to Braak stage, each case assigned an arbitrary case number (1-46), with gender; male (M) or Female (F), age in years, CERAD neuritic plaque scores, neuropathological observations, post-mortem interval (PMI) in hours and reported frontal cortical pH are provided for each case. – denotes data not available.

| **Antibodies** | **Epitope** | **Specificity** | **Dilution** | **Supplier** |
| --- | --- | --- | --- | --- |
| Tau | | | | |
| HT-7 | aa 159-163 | Pan-Tau | 1:5000 | Autogen Bioclear |
| AT8 | pSer199 + pSer202 + pThr205 | Phospho-Tau | 1:50 | Autogen Bioclear |
| PHF-1 | pSer396 + pSer404 | Phospho-Tau | 1:1000 | P.Davies Lab |
| CP13 | pSer202 | Phospho-Tau | 1:1000 | P.Davies Lab |
| Alz-50 | discontinuous epitope; aa7-9 and aa 312-342 | Conformational Tau | 1:1000 | P.Davies Lab |
| MC-1 | discontinuous epitope; aa7-9 and aa 312-342 | Conformational Tau | 1:1000 | P.Davies Lab |
| TOC1 | epitope preferentially exposed when oligomerized; aa209-244 | Oligomeric Tau | 1:5000 | N. Kanaan / L. Binder Lab |
| Amyloid | | | | |
| 6E10 | aa 3-8 of Aβ sequence | APP and metabolites including Aβ | 1:1000 | Biolegend |
| BACE1 | aa surrounding 490 | BACE1 | 1:1000 | Cell Signalling |
| MOAB-2 | aa 1-4 of Aβ sequence | Aβ only | 1:1000 | Cambridge Bioscience |
| Pyro-Glu | aa 3-16, pyro-glu E3 | Pyro-glu E3 modified Aβ | 1:1000 | Biolegend |

**Supplemental Table 2. Antibodies**. Antibodies are listed according to tau and amyloid antigens, commercial name, epitope, specificity, dilution and supplier are given. For phospho-dependant antibodies detected phospho-residues of either Serine (Ser) or Threonine (Thr) are given. For all antibodies epitope is given based on amino acid (aa) sequence according to the longest isoform.
